# The anti-tumoral potential of the saporin-based uPAR-targeting chimera ATF-SAP

**DOI:** 10.1038/s41598-020-59313-8

**Published:** 2020-02-13

**Authors:** S. Zuppone, C. Assalini, C. Minici, S. Bertagnoli, P. Branduardi, M. Degano, M. S. Fabbrini, F. Montorsi, A. Salonia, R. Vago

**Affiliations:** 1 Urological Research Institute, Division of Experimental Oncology, IRCCS San Raffaele Scientific Institute, Milano, Italy; 2 Biocrystallography Unit, Division of Immunology, Transplantation, and Infectious Diseases, IRCCS San Raffaele Scientific Institute, Milano, Italy; 3 Dipartimento di Biotecnologie e Bioscienze, Università degli Studi di Milano-Bicocca, Milan, Italy; 4 MIUR, Italian Ministry of Instruction, University and Research, 20090 Monza, Italy; 5 Università Vita-Salute San Raffaele, Milano, Italy

**Keywords:** Protein delivery, Breast cancer, Drug delivery, Targeted therapies

## Abstract

The development of personalized therapies represents an urgent need owing to the high rate of cancer recurrence and systemic toxicity of conventional drugs. So far, targeted toxins have shown promising results as potential therapeutic compounds. Specifically, toxins conjugated to antibodies or fused to growth factors/enzymes have been largely demonstrated to selectively address and kill cancer cells. We investigated the anti-tumor potential of a chimeric recombinant fusion protein formed by the Ribosome Inactivating Protein saporin (SAP) and the amino-terminal fragment (ATF) of the urokinase-type plasminogen activator (uPA), whose receptor has been shown to be over-expressed on the surface of aggressive tumors. ATF-SAP was recombinantly produced by the *P*. *pastoris* yeast and its activity was assessed on a panel of bladder and breast cancer cell lines. ATF-SAP resulted to be highly active *in vitro*, as nano-molar concentrations were sufficient to impair viability on tumor cell lines. In contrast to untargeted toxins, the chimeric fusion protein displayed a significantly improved toxic effect in uPAR-expressing cells, demonstrating that the selective activity was due to the presence of the targeting moiety. Fibroblasts were not sensitive to ATF-SAP despite uPAR expression, indicating that cell-specific receptor-mediated internalization pathway(s) might be considered. The *in vivo* anti-tumor effect of the chimera was shown in a bladder cancer xenograft model. Current findings indicate ATF-SAP as a suitable anti-tumoral therapeutic option to cope with cancer aggressiveness, as a single treatment or in combination with traditional therapeutic approaches, to appropriately address the intra- and inter- tumor heterogeneity.

## Introduction

Current clinical protocols for the treatment of solid tumors are mostly based on surgical debulking, followed by radiation and chemotherapy according to tumor grade and stage. The drugs used in the different chemotherapeutic protocols predominantly include small molecules, such as cisplatin, taxol, doxorubicin and anthracyclines, which eventually aim to target rapidly growing cells, blocking cell proliferation and DNA replication. Albeit in some cases efficient in reducing or completely eradicating tumor mass, these types of therapies suffer from a lack of specificity and a poor solubility of the molecules in water, killing cells in a cell cycle dependent manner and causing an increased toxicity against normal tissues (e.g., bone marrow, intestinal epithelial cells and hair follicles) besides killing rapidly dividing cancer cells.

Targeted therapies have gained great attention in the last decades, as they opened up the possibility to specifically deliver drugs to diseased tissues and kill cancer cells without or minimally affecting healthy organs. The target specificity is addressed by the interaction of drugs with molecules, mainly proteins, exclusively expressed or overexpressed in cancer cells. Recently, the molecular diagnostics has rapidly improved, and more and more targeted therapeutic agents have been approved by the US Food and Drug Administration (FDA) across many cancer subtypes, providing new treatment options to patients who cannot receive traditional chemotherapy. These therapies include hormones, signal transduction inhibitors, gene expression modulators, apoptosis inducers, angiogenesis inhibitors, immunotherapies and toxin-based drugs^1^. Even if safer, these approaches are not free from drawbacks. In fact, patients can stop responding to the treatment via complicated, tumor specific mechanisms that result in alterations of the drug target, activation of pro-survival pathways and ineffective induction of cell death; overall, that may be defined as multidrug resistance^2^. Toxins used as effectors in terms of cancer therapy have been studied for decades with promising results mainly in the form of immune conjugates. The presence of an antibody portion or ligand domain confers target specificity, providing an antigen-specific binding affinity to molecules over-expressed on the surface of malignant cells. One of the most successfully-developed toxin-based drug is Denileukin diftitox, the first recombinant protein which combines a truncated form of diphtheria toxin (DT) and human interleukin-2; the compound has been approved by the FDA for the treatment of cutaneous T-cell lymphomas. Numerous other toxin-based conjugates are under investigations in pre-clinical and clinical trials against hematological as well as solid tumors with encouraging results^3^. Main obstacles to a successful treatment of solid tumors with immunotoxins (ITs) include the poor penetration rate into tumor masses and the activation of the immune response against the antibody component, which eventually limits the number of treatment cycles that a patient may receive. In addition, even if the issue related to the size of chimeras has been overcome by using only the small variable fragment(s) of the antibody, the costs and scaling-up of production of these compounds remain a limitation.

Among toxins, type I plant-derived Ribosome-Inactivating Proteins (RIPs) belong to the N-glycosidase family of toxins able to depurinate a specific adenine base located in the universally conserved “GAGA” tetraloop in the 23/26/28S rRNA. This activity results in the inability of the ribosome to bind the elongation factor 2 and, thus, in the irreversible inactivation of protein translation, causing cell death by apoptosis^3,4^. The lack of a specific binding domain in type I RIP’s structure has allowed their direct exploitation as catalytic moiety of recombinant chimeras. In this class of molecules, SAP is one of the most studied because of its strong intrinsic RIP activity both in cell-free systems and in cell lines and its unusual resistance to high temperature, denaturation and attacks by proteolytic enzymes, making it a suitable candidate for therapeutic intervention. Its internalization pathway has been demonstrated to involve the low-density lipoprotein receptors (LDLR) family, that includes seven closely related family members: the very-low-density lipoprotein (VLDL) receptor, apoE receptor 2, multiple epidermal growth factor-like domains 7 (MEGF7), glycoprotein 330 (gp330/megalin/LRP2), lipoprotein receptor-related protein 1 (LRP1), and LRP1B^4^. Among these, the α2-macroglobulin receptor/low-density lipoprotein receptor-related protein 1 (LRP1) in particular was shown to bind saporin (SAP) *in vitro*^5,6^, mediating SAP internalization in human monocytes and fibroblasts^7,8^.

The urokinase plasminogen activator receptor (uPAR) represents a good candidate as a target for innovative cancer therapies. It is a glycoprotein of 55–60 kDa, associated to the external surface of the plasma membrane by a glycosyl-phosphatidylinositol (GPI) anchor and is an important regulator of the plasminogen activation system (PAS), an extracellular proteolytic cascade responsible for the extracellular matrix (ECM) degradation. Upon binding to its natural ligand, the urokinase plasminogen activator (uPA), uPAR allows the conversion of plasminogen into the serin-protease plasmin, which reciprocally cleaves and activates pro-uPA and matrix metallo proteases (MMPs), thereby facilitating cell migration by tissue remodeling. Thus, uPAR plays a pivotal role in microenvironment modification, promoting ECM proteolysis and ECM interaction. It participates in a number of transitory physiological processes that require blood vessels formation and ECM degradation (e.g., menstrual cycle and trophoblast implantation, embryonic development, inflammation and wound healing), being expressed by cells that are in motion, such as activated leukocytes, endothelial cells and fibroblasts^9^. However, uPAR expression is significantly upregulated in cancer under particular stimuli (such as hypoxia), where the reorganization of tumor microenvironment leads to cancer cells migration, angiogenesis, vascular invasion and propagation to distant sites. In agreement with its roles in cancer progression, the importance of uPAR is widely strengthened by a positive correlation between its levels and patient outcome/low overall survival in most human solid and hematological malignancies, including breast^10^ and bladder cancers^11^. The first uPAR targeting peptide was created by isolating the amino-terminal fragment (ATF) of uPAR natural ligand, uPA, containing the initial 135 amino acids including the growth factor domain^12,13^. ATF was shown to be highly efficient in the *in vitro* specific delivery of a biological toxic compound to uPAR overexpressing hematological tumors^6,13^.

Taking together all previous observations, we used ATF-SAP, a uPAR targeting chimera^6^, to assess whether ATF is able to specifically recognize and bind uPAR overexpressing cells, thus modifying the internalization pathway of the toxin and making it dependent on the expression of the target molecule. In this regard, we explore the co-expression of potential uPAR partner molecules in order to better understand ATF-SAP internalization pathways. Likewise, we also studied if SAP-based chimera is able to penetrate the tumor mass, mediating antitumor effects *in vivo*.

## Results

### uPAR over-expression associates with a stage II muscle invasive bladder cancer and triple negative breast cancer phenotypes

**Figure 1:**
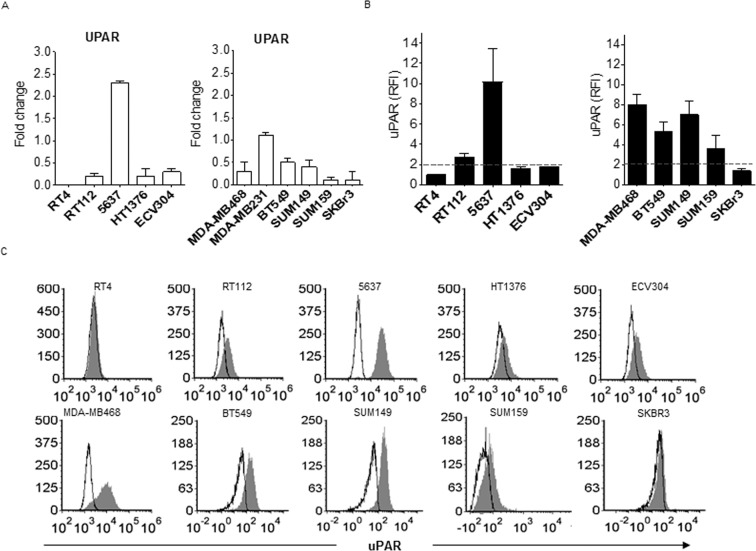
UPAR expression in bladder and breast cancer cell lines. Bladder and breast cancer cell lines resembling respectively different grades (RT4 -grade 1-, RT112 and 5637- grade 2-, HT1376 and ECV304 -grade 3-) and subtypes (MDA-MB-468, BT549, SUM149, SUM159 -TNBC-, SKBR3 -HER2^+^-) were analyzed by quantitative PCR (see “Materials and Methods”) for UPAR gene expression (**A**) and by flow cytometry for uPAR protein expression (b and c). UPAR protein expression is shown as histogram plots (**B**) and Relative Fluorescence Intensity (RFI) (see “Materials and Methods”) (**C**). SKBR3 HER2^+^ breast cancer cell line was used as subtype control. The dashed line represents the threshold arbitrarily defining positive expression (RFI = 2).

uPAR is known to exert an important role in tumor progression; there are many evidences of the positive correlation between uPAR levels and patient poor prognosis and overall survival^11^. With the aim to verify the clinical relevance of uPAR as a target, we firstly analyzed its gene and protein expression on a panel of bladder and breast cancer cell lines different in grading and invasiveness^14^ (Fig. 1). Heterogeneous levels of both transcript and protein were found in all the analyzed cell types: as regards for bladder cancer, a no or poor expression was detected in grade 1 (RT4) and grade 3 muscle invasive cells (HT1376, ECV304); conversely, a modest to evident overexpression was found in grade 2 bladder cancer cell lines (RT112 and 5637). On the other hand, among the TNBC cells analyzed, 3 out of 4 (MDA-MB-468, BT549 and SUM149) were highly uPAR positive, in contrast with HER2^+^ SKBR3 breast cancer cell lines.

### ATF-SAP chimera

After verifying the presence of uPAR on cancer cell lines *in vitro*, we took advantage of an uPAR-directed ATF-SAP chimera as a prototype of tumor targeting toxin^6,13^. This fusion protein is composed by SAP fused to the amino-terminal fragment (ATF) of human urokinase-type plasminogen activator (uPA), the natural ligand of uPAR. The insertion of a targeting moiety at the N-terminal of the toxin allows its delivery specifically to uPAR expressing cells, thus limiting potential off-target effects. ATF-SAP coding sequence was cloned into a pPICZ vector for yeast expression and its production and purification was optimized in the methylotrophic yeast *Pichia pastoris* and scaled up in bioreactors^6^. The yeast system was demonstrated to be a suitable platform for the expression of recombinant proteins according to Lombardi *et al*.^15^, allowing protein post-translation modifications, production and secretion of the toxic SAP chimera. A catalytically inactive mutant (ATF-SAP KQ) was produced as a control^6^.

### ATF selectively delivers SAP toxin to uPAR overexpressing bladder and triple negative breast cancer cells and induces apoptosis

**Figure 2:**
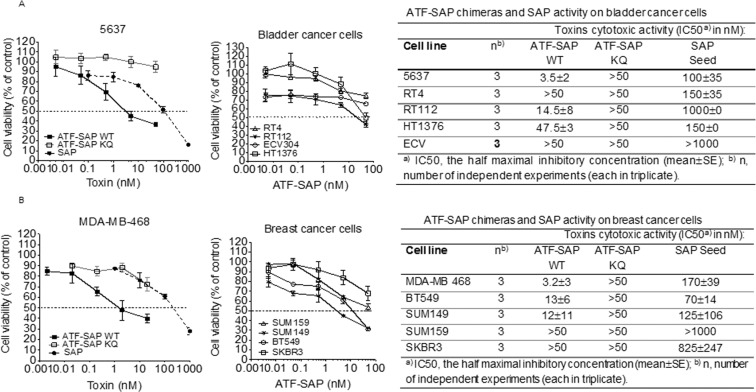
Cytotoxic activity of ATF-SAP on bladder and breast cancer cells. ATF-SAP activity and target specificity were evaluated on RT4, RT112, 5637, HT1376 and ECV304 bladder cancer cell lines (**A**) and on MDA-MB-468, SUM149, SUM159, BT549 TNBC and HER2^+^ SKBR3 breast cancer cell lines. (**B**) Cells were incubated for 72 h with scalar logarithmic concentrations of the toxin and cell viability was analyzed by MTT assay. The untargeted seed SAP and the catalytically inactive mutant ATF-SAP KQ were used as controls. The IC50 from three different experiments is reported as mean ± SE.

In order to characterize the *in vitro* biological activity of the uPAR targeting chimera, uPAR^+^ and uPAR^−^ bladder (Fig. 2A) and breast (Fig. 2B) cancer cell lines were incubated with scalar logarithmic concentrations of the toxin. As expected, ATF-SAP WT efficiency in killing cells was proportional to uPAR levels, impairing cell viability in a dose-dependent manner and in a higher significant extent compared to seed SAP, the untargeted control. In addition, cell death was unambiguously due to the presence of SAP enzymatic activity, as its catalytically inactive mutant ATF-SAP KQ failed to exert any effect. Accordingly, it is worth of noticing that ATF-SAP WT was not able to kill cells expressing low or undetectable uPAR levels (grade 1 and 3 bladder cancer and HER2^+^ breast cancer cell lines), meaning that a higher concentration of chimera is needed to reach an IC50, which results in a loss of receptor specificity. On the basis of these results, we can conclude that grade 2 bladder cancer and TNBC represent good models to test ATF-SAP biological activity; furthermore, ATF targeting domain is absolutely required to increase the toxin selectivity on uPAR^+^ cells in *in vitro* assays.

**Figure 3:**
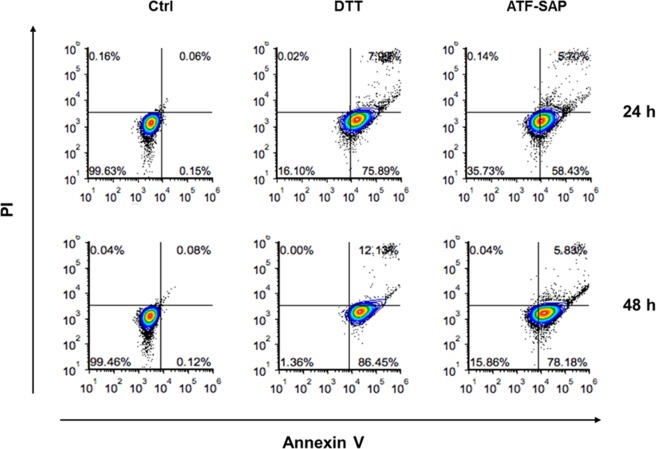
ATF-SAP cell apoptosis induction. 5637 bladder cancer cells were incubated with ATF-SAP for 24 or 48 hours. Flow cytometry analysis was performed to distinguish early apoptotic (lower right gate) form late apoptotic (upper right) and necrotic (upper left) cell populations.

It is well known that SAP toxin induces cell apoptosis. For corroborating evidence, we investigated the activation of programmed cell death by analyzing phosphatidylserine exposure in cells treated with ATF-SAP. To this aim, 5637 cells were incubated with ATF-SAP and apoptotic cell death was detected by flow cytometry (Fig. 3). At 72 hours we detected a significant population of cells undergoing late apoptosis driven by caspase 3 processing, which was already detectable 48 hours after incubation with the toxin (Fig. 3B).

### ATF-SAP internalization route is cell specific

**Figure 4:**
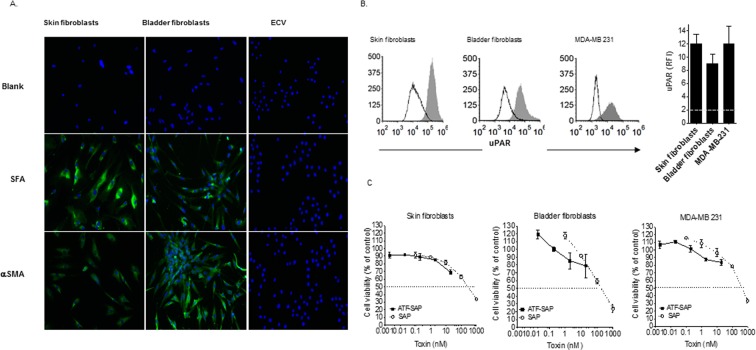
ATF-SAP activity on fibroblasts and MDA-MB-231 cancer cell line. (**A**) Molecular profile of skin and bladder-derived fibroblasts. Human skin- and bladder-derived primary fibroblasts were analyzed by immunofluorescence for the expression of Fibroblasts Surface Antigen (SFA) and alpha-Smooth Muscle Actin (αSMA). ECV304 epithelial bladder cancer cell line was used as negative control. Nuclei were stained with DAPI (blue). (**B**) uPAR expression on skin and bladder-derived fibroblasts as well as breast cancer MDA-MB-231 cell line was analyzed by flow cytometry and expressed as histogram plots (upper panel) and Relative Fluorescence Intensity (RFI) (see “Materials and Methods”) (lower panel). The dashed line represents the threshold arbitrarily defining positive expression (RFI = 2). (**C**) ATF-SAP toxic activity was evaluated after 72 h incubation and compared to the untargeted seed SAP. Results from one representative experiment are shown as mean ± SD. Three independent experiments were performed for each assay.

Next, we investigated the potential off-tumor toxicity of ATF-SAP by exploiting a non-tumoral cell line, such as healthy human skin derived fibroblasts. Due to their implication in physiological wound healing processes, fibroblasts are expected to express uPAR on their surface. In fact, as shown in Fig. 4A, high levels of uPAR were detected on these cells. Therefore, we wondered whether they were also sensitive to the activity of ATF-SAP. Notably, fibroblasts viability resulted unaffected by the toxin (Fig. 4C). To further corroborate their lack of sensitivity to the chimera and to validate bladder cancer as a candidate target for the proposed therapy, we extracted primary bladder-derived fibroblasts from a human bladder biopsy and, after immune-fluorescence characterization for typical fibroblasts markers, analyzed them for uPAR expression (Fig. 4A,B). Similarly, even if a high uPAR positivity could be detected, bladder fibroblasts were spared by ATF-SAP, that resulted completely ineffective (Fig. 4C). This unexpected behavior was also noticed on MDA-MB 231 breast cancer cell line, which, in spite of displaying a high uPAR expression, were not sensitive to the activity of ATF-SAP (Fig. 4C). These data suggest that in some cases uPAR expression is not the sufficient condition to mediate ATF-SAP toxicity.

### ATF-SAP internalization does not rely on uPAR natural ligands nor on uPAR cofactor LRP1

**Figure 5:**
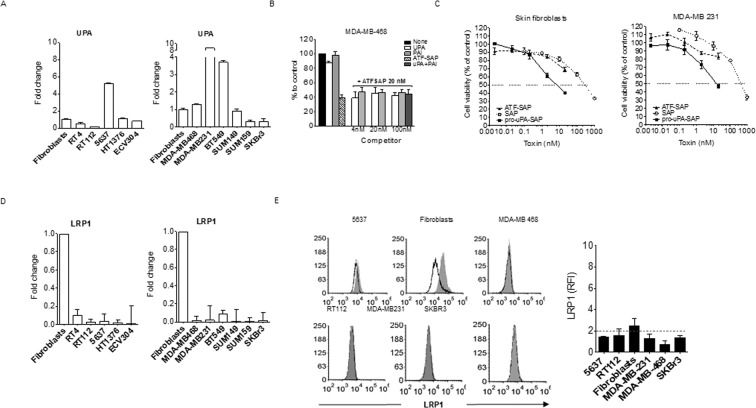
UPA and LRP1 expression in bladder and breast cancer cell lines. (**A**) Skin fibroblasts, bladder and breast cancer cell lines respectively resembling different stages (RT4 -T1 superficial-, RT112 and 5637- T2 muscle invasive-, HT1376 and ECV304 -T3 muscle invasive-) and subtypes (MDA-MB-468, MDA-MB-231, BT549, SUM149, SUM159 -TNBC-, SKBR3 -HER2^+^-) were analyzed by qPCR for UPA gene expression (see “Materials and Methods”). (**B**) Competition assay between ATF-SAP and uPAR natural ligands. MDA-MB-468 cells were incubated with ATF-SAP 20 nM in the presence of equal or increasing concentrations of uPA or PAI. The effect on cell viability was evaluated after 72 h by MTT assay. Seed SAP was used as untargeted control. (**C**) Comparison of pro-uPA-SAP and ATF-SAP toxic activity on fibroblasts and MDA-MB-231 cells. Results from one representative experiment are shown as mean ± SD. Three independent experiments were performed for each assay. LRP1 gene (**D**) and protein (**E**) expression on bladder and breast cancer cells. LRP1 protein expression is displayed as histogram plots (**E**, left panel) and RFI (see “Materials and Methods”) (**E**, right panel). The dashed line represents the threshold arbitrarily defining positive expression (RFI = 2).

Hence, we investigated if the amount of uPA produced by the same cell types is able to sequester its receptor from the ATF-SAP recognition and binding. To achieve this goal, we firstly analyzed its mRNA levels (Fig. 5A), but no differential uPA expression between ATF-SAP responding and not responding cells could be detected. Moreover, to deeper clarify whether high levels of uPA would compete with ATF-SAP for receptor binding and reduce the toxin uptake, we assessed the ability of ATF-SAP to be internalized in the presence of increasing concentrations of uPA and uPA inhibitor plasminogen activator inhibitor (PAI-1). The latter has been demonstrated to covalently bind and inhibit the proteolytic activity of uPAR-bound uPA, promoting LRP1 association and internalization of the complex^7^. As shown in Fig. 5B, ATF-SAP cytotoxicity remained unchanged, meaning that there is no competition with uPA for receptor binding. Finally, as a proof of concept, aiming at demonstrating that ATF-SAP endocytosis relies on a peculiar uPA:PAI-1-LRP1-independent pathway, we used, for internalization comparison, a SAP-based compound in which SAP is chemically conjugated to the entire prouPA ligand (pro-uPA-SAP)^16^. In this setting, both fibroblasts and MDA-MB-231 were efficiently killed by the conjugation product, indicating that uPAR can be efficiently bound and internalized in cells refractory to ATF-SAP (Fig. 5C).

Next, we sought to determine if some other factor(s) implicated in the urokinase plasminogen activation system may also be involved in toxin internalization. To this purpose, we initially investigated LRP-1 (low density lipoprotein receptor related protein 1), known to be important not only in uPAR endocytosis and turnover on cell surface but also for SAP receptor mediated internalization^5,7,16^. Unexpectedly, we could observe LRP1 to be highly expressed at an mRNA level in fibroblasts, but not in MDA-MB-231 cells (Fig. 5D). Analogously, we found ATF-SAP sensitive cells to have very low or even undetectable LRP1 levels. In order to exclude any post transcriptional regulation of the above-mentioned transcript, we also confirmed this result by measuring LRP1 at a protein level by flow cytometry analysis (Fig. 5E). Such observation suggests that LRP1 seems not to be an essential cofactor for ATF-SAP-bound uPAR internalization in these cells.

### Evaluation of uPAR expression stability and ATF-SAP activity on stage II bladder cancer cells after implantation in mice

**Figure 6:**
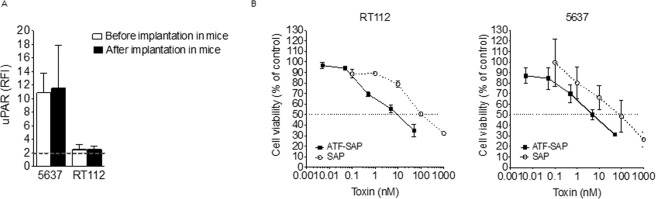
Cytotoxic activity of ATF-SAP on *ex vivo* bladder cancer cells. (**A**) After the second implantation, established tumors were minced and tumor cells analyzed for uPAR expression by flow cytometry. Results are shown as RFI (see “Materials and Methods”) (left panel). The dashed line represents the threshold arbitrarily defining positive expression (RFI = 2). (**B**) Explanted cells were then incubated for 72 h with ATF-SAP and cell viability was evaluated by MTT assay (right panels). Seed SAP was used as untargeted control. Results from one representative experiment are shown as mean ± SD. Three independent experiments were performed.

Since tumor propagation via prolonged cell culture may promote loss of surface antigens, we investigated if uPAR expression is stably maintained even with multiple serial tumor implantations in mice, in order to predict ATF-SAP efficiency in an *in vivo* environment. To this aim, grade 2 bladder cancer cells were subcutaneously injected and grown in the flank of NSG mice for two times and, once explanted, analyzed for uPAR expression and ATF-SAP sensitivity. As shown in Fig. 5, a comparable expression of the receptor was observed (Fig. 6A) and ATF-SAP dose-dependent biological activity was in line with previous results (Fig. 6B), further supporting the use of grade 2 bladder cancer cell lines as suitable *in vivo* models to test the chimera pharmacological efficacy.

### ATF-SAP antitumor activity in a subcutaneous model of bladder cancer

**Figure 7:**
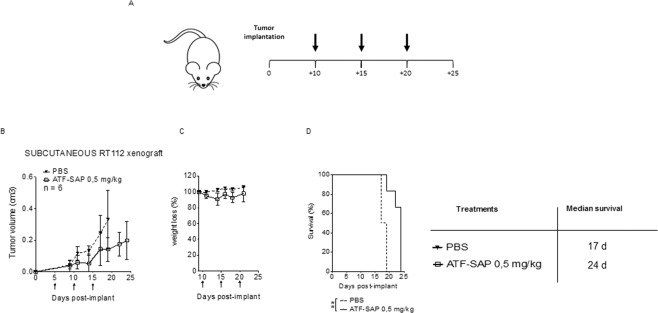
Effects of ATF-SAP on tumor growth in a subcutaneous xenograft bladder cancer mouse model. (**A**) Schematic representation of the experimental design. RT112 bladder cancer cells were subcutaneously injected in 7-week-old nude female mice. Tumor bearing mice were randomized into 2 experimental groups and treated respectively with PBS or ATF-SAP (0.5 mg/Kg) via intravenous injection every 5 days after tumor implantation. (**B**) Quantitative analysis of growing tumor volume in mice are shown as mean ± SD from respectively n = 6 mice per condition. (**C**) The mean weight of mice from each treatment group is shown as percentage from initial. (**D**) Kaplan–Meyer plot of animal survival with median survival time listed in the table. Results from a Mantel–Cox two-sided log-rank test are shown when statistically significant (**p < 0.01) for ATF-SAP 0.5 mg/kg (red; hazard ratio, 19.2; 95% CI, 2.2–165.8) versus PBS in mice bearing subcutaneous RT112 tumor.

We demonstrated that grade 2 bladder cancer cell lines RT112 and 5637 maintain a stable uPAR expression, even after serial implantation cycles in mice, representing good models to test the *in vivo* activity of the chimeric toxin. Thus, as a next step, we validated the therapeutic potential of ATF-SAP in a subcutaneous model of bladder cancer. To do so, RT112 cells were injected in the left flank of immunocompromised nude mice. After verification of tumor engraftment, 10 days tumor-bearing mice were intravenously administered with ATF-SAP in a dose equivalent to 0.5 mg/kg every 5 days for three times and monitored for toxic effects (Fig. 7A). As shown in Fig. 7B, tumor growth in mice treated with ATF-SAP was superimposable with controls (Fig. 7B); however, mice survival was significantly improved (Fig. 7D). Additionally, no signs of lethargy or weight loss were detected (Fig. 7C). Clearly, these results point out anti-proliferative effects of ATF-SAP.

## Discussion

**Figure 8:**
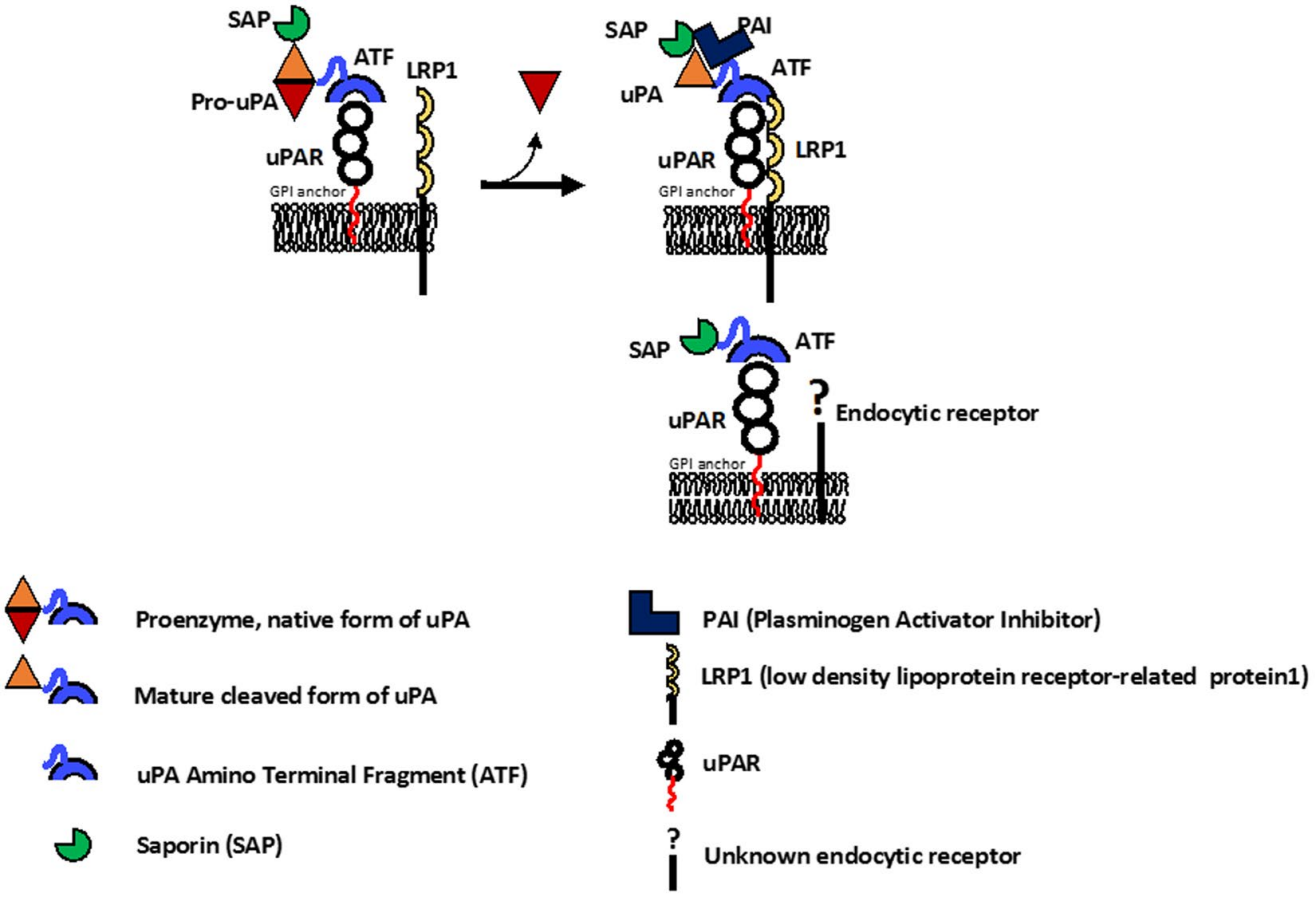
Schematic representation of internalization pathways proposed for uPAR targeting toxins. (**A**) LRP1-uPA-PAI dependent endocytosis; (**B**) LRP1-uPA-PAI independent endocytosis.

The production of recombinant proteins has gained great interest in the biopharmaceutical sector as an increasing amount of protein drugs are currently undergoing preclinical and clinical studies or have been already approved to be on the market. Unlike many chemical drugs, proteins are difficult to synthetize *in vitro* due to their complex structure, which is strictly related to their function. Therefore, host organisms are usually exploited to produce them throughout biotechnological processes. The choice of the most suitable expression host represents a key step in the development of this kind of drugs and relies on both the chemical and biological properties of the recombinant protein of interest. If complex post-translational modifications (N-glycosylation, phosphorylation, etc.) are required to ensure proper folding, necessary to guarantee the protein’s correct biological function, then an eukaryotic system is usually preferred to bacteria. This aspect is even more relevant for toxins, considering that they can be lethal for producing hosts as well. In the present study, we used ATF-SAP, a recombinant protein in which the toxin SAP is fused to the amino-terminal fragment (ATF) of the urokinase plasminogen activator, uPA^6,13,15^. Due to the length and structure of the ATF moiety (a 151 amino acids long peptide, comprising an EGF-like domain or growth factor domain-amino acids 12–32- followed by a kringle domain containing six disulphide bonds), ATF-SAP production was set up in *Pichia pastoris* yeast, in order to both fulfil a proper folding and minimize the toxicity for the host organism by directing the toxic fusion proteins to the secretory pathway. Thus, ATF-SAP expression has been optimized in a fermentation process using lab-scale stirred bioreactor to produce discrete batches and demonstrated to be actively cytotoxic on uPAR overexpressing U937 leukemia cells^6^. The choice of uPAR as a target arises from the clinical evidence of a positive correlation between uPAR expression levels and patient low overall survival in a variety of epithelial and hematological tumors, as it is involved in many steps of tumor progression. Hence, it provides the unique opportunity to tackle with multiple tumors with a single SAP-based product. As a proof-of-concept, a panel of human cell lines resembling bladder and breast cancer was analyzed for uPAR expression in order to identify the best model to test ATF-SAP activity and specificity. We found that increased levels of this receptor were present in high-grade urothelial bladder cancer lines and TNBC phenotype, which therefore were selected to be suitable *in vitro* models, and ATF-SAP was proven to recognize and kill uPAR-positive cells, proportionally to its expression, by inducing apoptotic cell death. As a corollary, cell lines lacking the receptor are not sensitive to the chimera, supporting the hypothesis that the presence of ATF enhances an efficient tumor-targeted delivery of SAP activity. In clinical trials, the pharmacological efficiency of current targeted therapies in the treatment of tumors usually associates with toxicities deriving from off-tumor expression of the target. Besides its implication in cancer evolution, uPAR plays a key role in pericellular proteolytic activity, related to tissue remodeling during normal physiological condition, such as wound healing and initiation of angiogenesis^9,17^. Since fibroblasts are the most involved cell type in the former cited process, we analyzed ATF-SAP activity on a skin fibroblast cell line as well as primary bladder derived fibroblasts, in order to predict any potential *in vivo* side effect. Even if both types of fibroblasts are highly uPAR expressors, ATF does not determine any increase of the SAP activity on these cells, failing to exert a cytotoxic effect at the given concentrations. Unexpectedly, we also observed that ATF-SAP lacked activity toward the highly uPAR-positive MDA-MB-231 TNBC cell line. The reason for such behavior could be explained by a differential expression of accessory mechanisms/molecules involved in the receptor internalization in highly susceptible tumor cells. To deeper understand this phenomenon, we focused on the principal members of the plasminogen activation system. It is well known that uPAR endocytosis and recycling on the plasma membrane directly relies on different components, among which its natural ligand uPA (responsible for the activation of plasminogen cascade), the plasminogen activator inhibitor-1 (PAI-1) and the alpha2-macroglobulin receptor/low-density lipoprotein receptor-related protein (LRP-1). When uPA associates with uPAR, it is converted into the active two-chain form, which reacts rapidly with PAI-1, forming a complex that is bound and internalized by LRP1. This route leads to the degradation of the ligand and its inhibitor and to the turnover of the receptor on the cellular surface, allowing the attenuation of proteolytic processes in the extracellular environment^18,19^. As the activity of the chimera was evident in almost all the uPAR expressing cell lines in our hands, we wondered if there were some differences in the regulation of LRP1 co-receptor or natural ligand uPA compared to fibroblasts and MDA-MB-231 cells. Notably, previous studies demonstrated that SAP binds to LRP1 *in vitro*, resulting in its internalization in human monocytes and in fibroblasts, further strengthening the importance of this receptor in this complex system^5^. In 1997, Fabbrini *et al*.^13^ demonstrated that LRP1 is strongly required in ATF-SAP endocytosis. In fact, the observation of toxin resistance in LRP1-deficient cells and competition of anti-LRP1 antibodies to ATF-SAP cytotoxicity, led to the conclusion that ATF might be necessary for uPAR targeting, but the internalization is due to the binding of the toxin to LRP1^4,16^. Nevertheless, when we analyzed the LRP1 gene expression and protein localization on the plasma membrane, we confirmed its high levels on fibroblasts, but not on MDA-MB-231 nor on uPAR^+^ ATF-SAP sensitive cells, suggesting that the SAP-based chimera internalization does not depend on this co-receptor. Similarly, the abundant uPA expression in both responding and non-responding cells and the absence of a competitive activity with ATF-SAP, excluded the implication of the natural ligand in sequestering uPAR, thus reducing its availability on cell surface. It is also worth of noting that ATF-SAP only contains the amino terminal binding domain of uPA, which is not expected to be able to bind PAI-1 and form the complex necessary for the internalization. Indeed, no changes in the ATF-SAP toxic activity were detected when PAI was added to the medium. On the other hand, when we used a SAP-prouPA conjugate carrying the entire pro-uPA zymogen sequence as a targeting moiety, the activity of SAP was greatly increased in both fibroblasts and MDA-MB-231 cell lines not responding to ATF-SAP. This observation suggests that different mechanisms might be involved in the internalization of the two SAP-based products. In 2008, Cortese *et al*. described a novel unusual internalization pathway responsible for constitutive endocytosis of uPAR that partially ties with macropinocytosis^20^. They observed that this so far unknown endocytosis pathway does not require the presence of uPA, PAI or LRP1 nor is related to uPAR massive expression on cell surface, as it could be detected either on naturally uPAR expressing cells and on cells transfected to express exogenous uPAR, but showing low uPAR levels. Apparently, this macropinocytosis-like mechanism is also clathrin- and lipid rafts- independent and associates to a rapid recycling of the receptor on the cell surface, therefore it might be important in unknown regulations of extracellular functions under particular conditions (i.e. in the absence of ligands or inhibitors). Up to now, macropinocytosis is a still poorly understood mechanism, constitutively induced in some cell types (e.g. dendritic cells and macrophages), but also strictly regulated in cells stimulated with growth factors (such as EGF)^20,21^. In our study we could distinguish different conditions, which might indicate different internalization pathways: uPAR^+^ LRP1^−^ MDA-MB-468, 5637 and RT112 cells were responsive to ATF-SAP; uPAR^+^ LRP1^−^ MDA-MB-231 cells were not sensitive to ATF-SAP, but turned to a restored SAP toxicity when pro-uPA-SAP was instead added; uPAR^+^ LRP1^+^ fibroblasts were not responsive to ATF-SAP, but became similarly sensitive to pro-uPASAP. We therefore may propose a model involving two different internalization routes for either ATF-SAP chimera or pro-uPA-SAP conjugate: an LRP1 and ligand-independent endocytosis, induced by the ATF binding to uPAR, mainly detected on LRP1 negative cancer cells, but not on MDA-MB-231; an LRP1 and ligand-dependent endocytosis, responsible for the pro-uPA-SAP endocytosis observed in LRP1 positive fibroblasts (Fig. 8). The latter could be explained as the need of fibroblasts to activate the plasminogen activation system in physiological conditions (such as wound healing process) and uptake the proteolytic ligands (uPA) in order to attenuate the extracellular process. On the other hand, this scenario does not elucidate the lack of cytotoxicity by ATF-SAP in LRP1 negative MDA-MB-231 cells that, on the contrary, are sensitive to pro-uPA-SAP.

So, ATF-SAP toxic activity in this system does not rely on the presence of the uPA:PAI ligands or LRP1 internalization cofactor, but it is likely able to stimulate a different micropinocytosis-like process that is supposed to faster uPAR turnover on the cell membrane. Whether this kind of micropinocytosis is a cell-specific mechanism or if there are other cofactors involved in the intracellular sorting machinery that might end up in the lysosomal degradation of ATF-SAP are still open questions that require further investigation.

Intra-tumor heterogeneity represents one of the major obstacles for targeting therapies, together with the ability of tumor cells to suppress effector response by down-regulating surface antigens. In order to face this issue, we firstly validated the effect of an *in vivo* environment on the expression of uPAR receptor. RT112 and 5637 bladder cancer cell lines were used as models, due to their differential expression of uPAR. We observed that after serial implantations in mice, both cell lines maintain uPAR levels and responsiveness to ATF-SAP, further confirming the suitability of this GPI anchored molecule as a target. When we tested ATF-SAP in an allogenic mouse model of bladder cancer, we could appreciate a tumor growth delay and an increase of animal overall survival, without observing toxic effect as weight loss or lethargy. This indicate that also *in vivo* ATF-SAP maintain its target selectivity and do not show adverse effects in the experimental conditions. Such treatment may represent an alternative to chemotherapy or may be used alone or in combination with other therapeutic options based on different mechanisms of action to better tackle cancer cells. The overall results confirm the suitability of SAP-based uPAR-targeting therapy and open the way to the validation of the chimeric toxin activity in other preclinical models.

## Materials and Methods

### Production and purification of ATF-SAP

The ATF-SAP production and purification were performed as previously described (Errico Provenzano *et al*. Micr. Cell Fact. 2016). Briefly, yeast cells expressing ATF-SAP were grown in BMGY medium [1% (w/v) yeast extract; 2% (w/v) peptone; 100 mM phosphate buffer, pH 6.0; 1.34% (w/v) yeast nitrogen base, 4 × 10^−5%^ (w/v) biotin; 4 × 10^−3%^ (w/v) histidine, 10 gr/l glycerol] in a 2 l stirred tank bioreactor (Braun Biotech, Melsungen, Germany) at 30 °C, pH 5.5 and stirring speed increasing upon oxygen consumption. After 24 h, when glycerol was totally consumed, 100 ml of methanol were added in a 16 h fed-batch phase. After the fermentation process yeast cultures were centrifuged at 10000 × *g* for 30 min at 4 °C and the supernatant containing ATF-SAP was passed through 0.22 µm, 100 nm and 10 nm filters. ATF-SAP was retained by the last filter, subjected to dialysis and further purified by ion exchange chromatography through CM Sepharose Fast Flow column (GE Healthcare) and eluted with PBS/1 M NaCl. Eluates were exchanged against PBS as above and filter sterilized.

### Cells culture

Human bladder RT4, RT112, 5637 (purchased in 2015 from Cell Lines Service), HT1376 (2015, SIGMA) and breast -MDA-MB 231, MDA-MB 468, BT549, SKBR3 (2015, ATCC) cancer cell lines were maintained in RPMI1640 (bladder) or DMEM medium (breast), respectively, supplemented with 10% FCS, 2 mM L-Glutamine and antibiotics (100 U/mL penicillin and 100 μg/mL streptomycine-sulphate). Human ECV304 (2015, Cell Lines Service) bladder cancer cells were maintained in medium 199 supplemented with 10% FCS, 2 mM L-Glutamine and antibiotics (100 U/mL penicillin and 100 μg/mL streptomycine-sulphate). Human SUM149 and SUM159 triple negative breast cancer cells (2015, Asterand Bioscience) were cultured in HAM-F12 medium, supplemented with 5% FCS, 2 mM L-Glutamine, antibiotics (100 U/mL penicillin and 100 μg/mL streptomycine-sulphate), insulin (2 μg/ml) and hydrocortisone (1 μg/ml), 10 mM HEPES. All the experiments were carried out between 3 and 15 passages after thawing. Mycoplasma test was performed monthly by PCR assay and only mycoplasma negative cells were used for the experiments. Pro-uPA-SAP was kindly supplied by Ugo Cavallaro^5^.

### Flow cytometry analysis

Cultured cell lines were detached by TripLE Express (Gibco) to preserve receptor integrity, washed with PBS containing 1% FCS and incubated with primary antibody (Ab) specific for human uPAR (Cat n° ADG3937, Lot AK647/01-1, Sekisui) or LRP1 (Cat n° 37-3800, Lot RH230932, Invitrogen). For uPAR detection, cells were incubated with the primary Ab at 4 °C for 1 h, followed by secondary antibodies conjugated to FITC for 30 min. Stained cells were re-suspended in 100 μL of PBS containing 1% FCS. Secondary FITC-conjugated antibody only or isotype control stained cells were used as negative controls. Samples were run through an AccuriTM flow cytometer (BD Biosciences). All data were analysed by FCS Express and expressed as relative fluorescence intensity (RFI), calculated as follows: mean fluorescence intensity after mAb staining/mean fluorescence intensity after isotype-negative control staining. 5637 and RT112 cells were cultured in 24-well microplates until 60% confluence. 24 hours later, the cells were treated with labelled exosomes (15 μg/well) and incubated at 37 °C, 5% CO2 for 48 hours. Then, cells were detached by incubation with 0.01% trypsin for 5 min at 37 °C, collected, washed with 1% FBS (in PBS) and subjected to flow cytometry analysis using AccuriTM flow cytometer. Analysis was done on 20,000 gated events per sample.

### MTT assay

Cultured cell lines were seeded in 96 wells plates and incubated for 72 hours with scalar logarithmic concentrations of ATF-SAP WT, ATF-SAP KQ, pro-uPA-SAP, SAP alone (seed SAP). After 72 hours of incubation at 37 °C, 3-(4,5-dimethylthiazol-2-yl)-2,5-diphenyltetrazolium bromide (MTT) (5 mg/ml in PBS) was added (0.5 mg/ml working concentration). After 1-hour incubation at 37 °C, supernatants were removed and 100 μl/well of dimethyl sulfoxide were added to dissolve formazan crystals. Cell viability was assessed by measuring the absorbance at 570 nm and expressed as percentage to untreated control. The toxic activity of the recombinant proteins was evaluated as IC50, shown as a mean ± SD from three independent experiments. For the competition experiments, MDA-MB-468 cells were seeded in 96 well plates, exposed to 20 nM ATF-SAP in the absence or presence of increasing concentrations of uPA (4, 20, 100 nM), PAI (4, 20, 100 nM) or uPA + PAI (100 nM + 100 nM), for 72 hours. Residual cells viability was evaluated by MTT assay.

#### Apoptosis cell death detection

5637 bladder cancer cell line were seeded in 24 wells plates and incubated with ATF-SAP 2 nM. After 72 hours of incubation at 37 °C, cells were harvested, washed in PBS and the viability rate was quantified by flow cytometry by using FITC-Annexin V/propidium iodide (PI) labeling assay (FITC Annexin V/Dead Cell Apoptosis Kit, Invitrogen, Catalog no. V13242). Cells cultured in the absence of ATF-SAP toxin were used as negative control. Flow cytometry was performed on Accuri ^TM^ flow cytometer (BD Biosciences) and data were analyzed using FCSExpress6 software.

### Establishment of primary human bladder-derived fibroblasts culture

Human bladder biopsies were obtained from men undergoing surgical resection. All the studies carried out on patient’s samples were approved by the Institutional Ethical Committee (Ospedale San Raffaele, Milan, protocol URBBAN, Rev. February 2nd, 2014, approval date March 3rd, 2014) and the specific informed consent was obtained. All the experimental procedures involving human biologic material were carried out in compliance with relevant guidelines and regulations. Cells were obtained through a primary explant technique. Briefly, the tissue was minced with a scalpel into small pieces (1–2 mm^3^) and washed in fresh medium to eliminate the excess of mucus or blood proteins. Tissue pieces were then placed on a dish for primary culture and incubated in MEM-Non-essential amino acids (100× , without L-Glutamine) (Lonza), supplemented with 1% FCS, antibiotics (100 U/mL penicillin and 100 μg/mL streptomycine-sulphate), 200 nM Hydrocortisone (Sigma), 10 nM Triiodothyronine (Sigma), 5 ng/ml Epidermal growth factor (Gibco, Life Technologies). The incubation was carried out for 3–5 days. Then the medium was changed at weekly intervals until a substantial outgrowth of cells was observed. Thus, the explants were removed and the cells transferred to a fresh dish. Cells were cultured for a maximum of 20 days.

### Immunofluorescence analysis

ECV304 bladder cancer cells, skin and bladder-derived fibroblasts were seeded in sub-confluent conditions and grown overnight on glass coverslips. After 24 hours, cells were quickly washed in PBS and fixed in 4% paraformaldehyde in PBS at room temperature for 30 min. After extensive PBS washing to remove the excess of paraformaldehyde, cells were permeabilized in 0.2% Triton and incubated in 10% donkey serum in PBS for 1 hour. Primary antibody incubation was performed for 1 hour at room temperature using: anti-human Fibroblasts Surface Protein (Cat n° ab11333, Lot GR65032-16, Abcam, working dilution 1:100 in 1% donkey serum, 0.1% Triton in PBS) or anti-human alpha-Smooth Muscle Actin (Cat n° A2547, Lot 032M4822, Sigma-Aldrich, working dilution 1:500 in 1% donkey serum, 0.1% Triton in PBS). Fluorescent signal was detected by the use of Alexa488-conjugated secondary antibody (working dilution 1:500 in 1% donkey serum, 0.1% Triton in PBS). Then, coverslips with stained cells were mounted on a microscope slide with VECTASHIELD Antifade Mounting Medium with DAPI (Vector Laboratories, Burlingame, CA, USA), carefully avoiding the formation of air bubbles. Fluorescent images were taken by using Axio Vision Imaging Software (Axiovision Rel 4.8®) on an Axio Imager M2 microscope (Carl Zeiss, Oberkochen, Germany).

### RNA extraction and quantitative analysis

Total RNA from bladder and breast cancer cell lines was extracted using TRIzol LS Reagent (Invitrogen) according to the manufacturer’s recommended protocols. The total RNA quantity was assessed using a Nanodrop spectrophotometer (ND-1000, Nanodrop, Labtech International, Uckfield, UK). Each RNA sample was then retro-transcribed with High Capacity cDNA Reverse Transcription Kit (Applied Biosystems). A calibration curve was performed for each gene of interest to test primers and to determine the correct concentration of cDNA to be used for quantitative Real Time. Each cDNA sample was diluted with nuclease free water to obtain the correct amount (0.5 ng) in a volume of 12.5 μl. The mix for the primers was prepared as follows: 12 μl of SYBR green PCR master mix and 0.5 μl of primers (forward and reverse). The total reaction was set for 25 μl/well. Reactions were run on ABI 7000 Real Time PCR, Applied Biosystems. Primers were purchased from Eurofins. UPAR, UPA and LRP1 expression levels (indicated as “fold change”) were analyzed in triplicate, normalized to HPRT1 and calculated according to the CT method. Primers used: UPAR-F 5′-GCCCAATCCTGGAGCTTGA-3′, UPAR-R 5′-TCCCCTTGCAGCTGTAACACT-3′; UPA-F 5′-TGAGGTGGAAAACCTCATCC-3′, UPA-R 5′-CTCCTTGGAACGGATCTTCA-3′; LRP1-F 5′–TAGACCGGCCCCCTGTGCTGTTGA-3′, LRP1-R 5′–GGTCTGCCGCGTGCTCGTAGGTGT-3′; HPRT1-F 5′-TGCAGACTTTGCTTTCCTTG–3′, HPRT1-R 5′- CTGGCTTATATCCAACACTTCG–3′.

### *Ex vivo* studies

A sub-confluent 5637 or RT112 bladder cancer cells were plated in 10 mm dishes, detached in exponentially growth conditions and re-suspended in serum and antibiotics free RPMI. 7-week-old NSG mice were subcutaneously injected with 1 × 10^6^ cells (RT112 or 5637) in the left flank. Tumor growth was monitored with caliper. Animals were sacrificed when the tumor started to grow exponentially. The tumor mass was explanted, minced and dissociated with Trypsin (0.25 mg/ml in RPMI) at 37 °C for 10 min to obtain single-cell suspension. Cells were then further mechanically disaggregated by pressing the pellet through a cell strainer (70 μm) and treated with DNAse (100 μg/ml, Sigma-Aldrich) and ACK lysing buffer (Lonza) to remove red blood cells. Single cells were finally washed extensively with medium supplemented with FCS 10%, 2 mM L-Glutamine and antibiotics (100 U/mL penicillin and 100 μg/mL streptomycine-sulphate) and dispersed into 10 mm dishes. After 1 week of *in vitro* culture, cells were collected, counted and reinjected in the left flank of 7-weeks old NSG mice (1 × 10^6^ cells/mouse). After mice sacrifice and cells recovery from tumor mass dissociation, flow cytometry analysis to monitor uPAR expression and cell-killing assay with ATF-SAP were performed as described above.

### *In vivo* studies

Studies on animal models were approved by the Institutional Animal Care and Use Committee (Institutional Animal Care and Use Committee, IACUC, approval number 901/2017-PR) and performed according to the prescribed guidelines and regulations. Subcutaneous xenograft mouse model: 7-week-old CD1 nude female mice (Charles River, Calco, Italy) were injected in the left flank with 1 × 10^6^ RT112 living cells; 10 days later, mice were treated with ATF-SAP in PBS solution (200 μl) administered intravenously (i.v.). The treatment was repeated every 5 days for 3 times. Subcutaneous tumor growth was monitored every two days by measuring tumor volumes with caliper as described previously. Tumor volumes are shown as mean ± SD (6 animals/group) and calculated as follows: r1 × r2 × r3 × 4/3 π, where r1 and r2 are the longitudinal and lateral radii, and r3 is the thickness of tumors protruding from the surface of normal skin. Mice weight was recorded every 2 days and their health/behaviors monitored daily. Animals were euthanized when tumors reached 10 mm in diameter, became ulcerated or a 20% weight loss was detected.

### Statistical analysis

All *in vitro* experiments were performed in triplicate. Mouse experiments were performed using 6 mice per treatment group. To compare tumor growth between the two groups, non-parametrical Mann-Whitney test was performed. Survival curves were compared using the log rank test.
